# Disrupted functional connectivity of the habenula links psychomotor retardation and deficit of verbal fluency and working memory in late‐life depression

**DOI:** 10.1111/cns.14490

**Published:** 2023-10-07

**Authors:** Ting Su, Ben Chen, Mingfeng Yang, Qiang Wang, Huarong Zhou, Min Zhang, Zhangying Wu, Gaohong Lin, Danpeng Wang, Yue Li, Xiaomei Zhong, Yuping Ning

**Affiliations:** ^1^ Department of Radiology The Affiliated Brain Hospital of Guangzhou Medical University Guangzhou China; ^2^ Geriatric Neuroscience Center The Affiliated Brain Hospital of Guangzhou Medical University Guangzhou China; ^3^ Guangzhou Medical University Guangzhou China; ^4^ Key Laboratory of Neurogenetics and Channelopathies of Guangdong Province and the Ministry of Education of China Guangzhou Medical University Guangzhou China; ^5^ The First School of Clinical Medicine Southern Medical University Guangzhou China; ^6^ Guangdong Engineering Technology Research Center for Translational Medicine of Mental Disorders Guangzhou China

**Keywords:** cognitive impairment, functional connectivity, habenula, late‐life depression, MRI, verbal fluency, working memory

## Abstract

**Background:**

Functional abnormalities of the habenula in patients with depression have been demonstrated in an increasing number of studies, and the habenula is involved in cognitive processing. However, whether patients with late‐life depression (LLD) exhibit disrupted habenular functional connectivity (FC) and whether habenular FC mediates the relationship between depressive symptoms and cognitive impairment remain unclear.

**Methods:**

Overall, 127 patients with LLD and 75 healthy controls were recruited. The static and dynamic FC between the habenula and the whole brain was compared between LLD patients and healthy controls, and the relationships of habenular FC with depressive symptoms and cognitive impairment were explored by correlation and mediation analyses.

**Results:**

Compared with the controls, patients with LLD exhibited decreased static FC between the right habenula and bilateral inferior frontal gyrus (IFG); there was no significant difference in dynamic FC of the habenula between the two groups. Additionally, the decreased static FC between the right habenula and IFG was associated with more severe depressive symptoms (especially psychomotor retardation) and cognitive impairment (language, memory, and visuospatial skills). Last, static FC between the right habenula and left IFG partially mediated the relationship between depressive symptoms (especially psychomotor retardation) and cognitive impairment (verbal fluency and working memory).

**Conclusions:**

Patients with LLD exhibited decreased static FC between the habenula and IFG but intact dynamic FC of the habenula. This decreased static FC mediated the relationship between depressive symptoms and cognitive impairment.

## Graphical abstract

Patients with late‐life depression (LLD) exhibited decreased static FC between the right habenula and bilateral IFG compared with healthy controls, and this decreased FC was involved in the underlying pathway connecting psychomotor retardation and deficit of verbal fluency and working memory. The present results provide a more in‐depth understanding of their pathological mechanism, and could inform the development of more targeted and effective therapeutic interventions, improving the management and treatment outcomes for LLD.

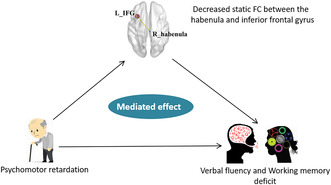

## 1 INTRODUCTION

Late‐life depression (LLD) is a detrimental mental health issue that affects 3.5%–7.5% of the elderly population,^1^ and it has been linked to an increased risk of mortality and dementia, imposing an enormous burden worldwide.^2^ Cognitive impairment is common in patients with LLD and can manifest as deficits in memory, attention, language, and executive dysfunction, all of which contribute to a reduced quality of life and poor clinical outcomes.^1^ Interestingly, many mechanisms are shared between depressive symptoms and cognitive impairment (such as a disrupted hypothalamic‐pituitary‐adrenal axis, neuroinflammation, and amyloid accumulation), which complicates the diagnosis and treatment of LLD patients.^3^ Therefore, exploring the underlying pathways linking depressive symptoms and cognitive impairment is highly important for understanding the pathological mechanism of LLD and may provide potential targets for precision interventions.

Over the past decade, the habenula has attracted increasing interest in the field of psychiatry. It is critical in the regulation of monoaminergic neurotransmission (particularly serotonin and dopamine), which is essential for emotion and cognitive regulation.^4^ Additionally, the habenula is implicated in the modulation of reward processing, stress responses, and aversive learning—all vital components in the development of depression. An increasing number of studies have revealed that the habenula is hyperactive in individuals with major depressive disorders (MDD), and inactivating the habenula by deep brain stimulation^5^, ^6^ or suppressing the burst firing of habenular neurons with ketamine^7^ can alleviate depressive symptoms, suggesting a potential causal relationship between habenular dysfunction and depressive symptoms. Additionally, disrupted functional connectivity (FC) of the habenula with other regions has been found in patients with MDD,^8^ subclinical depression,^9^ and treatment‐resistant depression,^10^ and this disruption was associated with the severity of depressive symptoms and suicidal ideation^11^ as well as the magnitude of the antidepressant effect of ketamine.^12^, ^13^

This evidence suggests that not only the activity but also the connectivity of the habenula is involved in the pathophysiology of depression. However, the specific relationship between habenular dysfunction and LLD remains underexplored. A previous study indicated that the extent of iron deposition in the habenula was correlated with cognitive scores in patients with LLD taking antidepressants,^14^ but whether patients with LLD exhibit functional abnormalities in the habenula remains unclear. Considering that the underlying pathophysiological mechanism of LLD may be different from that of adult depression,^2^ we hypothesized that LLD patients exhibit a different pattern of habenular FC than that observed in MDD patients. Additionally, the habenula plays a critical role in regulating working memory, and disconnection of the habenula and frontal cortex causes cognitive impairment.^15^ Considering that the connectivity between the habenula and frontal cortex is involved in affective and cognitive processing, we hypothesized that the FC between the habenula and frontal cortex mediates the association between depressive symptoms and cognitive impairment.

The present study aimed to investigate patterns of habenular FC in patients with LLD using static and dynamic FC analyses, and to explore the relationship of habenular FC with depressive symptoms and cognitive impairment. Because static FC reflects the average connectivity throughout the entire fMRI session and dynamic FC reflects the temporal variability in spontaneous fluctuations in connectivity,^16^, ^17^, ^18^ the present study applied both static and dynamic FC to fully elucidate the pattern of habenular FC in LLD patients. By identifying the neural mechanisms underlying LLD with a particular focus on the role of the habenula, the present study may enhance understanding of the pathophysiology of LLD and help identify potential neural correlates associated with depressive symptoms and cognitive impairments.

## 2 METHODS

### 2.1 Participants

A total of 202 individuals, comprising 127 patients diagnosed with LLD and 75 healthy controls (HCs), were enrolled in the study. The participants were recruited from both the community of Guangzhou and the Affiliated Brain Hospital of Guangzhou Medical University. Prior to participation, written informed consent was obtained from each participant or their legal guardians. The Ethics Committee of the Affiliated Brain Hospital of Guangzhou Medical University approved the study (Approval number: 2014, 078).

To be eligible for inclusion in the LLD group, participants were required to meet the following criteria: (1) aged 55 years or older and right‐handed; (2) met the Diagnostic and Statistical Manual of Mental Disorders, fourth edition (DSM‐IV), criteria for major depressive disorder with onset after the age of 55 years; and (3) received a clinical diagnosis and stage confirmation by trained physicians at the hospital. Healthy controls were included if they met the following criteria: (1) were right‐handed, (2) exhibited normal cognitive function, and (3) had no history of depression.

The exclusion criteria for patients with LLD and HCs were as follows: (1) presence of major psychiatric illnesses such as bipolar disorder or schizophrenia; (2) presence of physical ailments that may lead to mental abnormalities such as anemia or hypothyroidism; (3) major neurological conditions such as Parkinson's disease or stroke; (4) presence of metal implants or claustrophobia that could interfere with magnetic resonance imaging; or (5) history of psychotic symptoms, either past or present. The neuropsychologist and geriatric psychiatrist were responsible for conducting the diagnoses and assessments.

### 2.2 Clinical measurements

At the time of enrolment, demographic information, including sex, age, and education level, as well as clinical history information, such as illness duration and number of depressive episodes, was collected for all participants. The severity of depressive symptoms was evaluated using the 17‐item Hamilton Depression Rating Scale (HAMD‐17) and Geriatric Depression Scale (GDS). The five HAMD‐17 factors, namely, psychomotor retardation, cognitive bias, anxiety/somatization, sleep disturbance, and weight, were computed using the methodology described by Zhao et al.^19^ The assessments were carried out by two trained professional psychiatrists who had successfully completed a concordance assessment.

### 2.3 Neuropsychological assessments

Following standard clinical evaluations, participants underwent an interview with neuropsychologists to assess global cognitive function using the Mini‐Mental State Examination (MMSE). Subsequently, they completed a battery of neuropsychological tests to evaluate performance in five cognitive domains, including information processing speed (Symbol Digit Modalities Test (SDMT), Stroop Colour and Word Test Part A (Stroop A), and Trail‐Making Test Part A (TMT A)); memory (Auditory Verbal Learning Test (AVLT) and Working Memory Test (WMT)); language (Boston Naming Test (BNT) and Verbal Fluency Test (VFT)); executive function (Stroop Colour and Word Test C (Stroop C) and Trail‐Making Test Part B (TMT B)); and visuospatial skills (Rey‐Osterrieth Complex Figure Test (ROCF)).

### 2.4 MRI data acquisition

Following the neuropsychological assessments, the study participants underwent magnetic resonance imaging (MRI). Imaging data were acquired using a Philips 3.0 T MR system (Achieva, Netherlands) located at the Affiliated Brain Hospital of Guangzhou Medical University. An anatomical image was obtained with a sagittal 3D gradient echo for each participant. Resting‐state fMRI data for the entire brain were acquired using a single‐shot gradient echo‐planar imaging pulse sequence, with a total acquisition time of 8 min. The scanning parameters for resting‐state fMRI were as follows: TE = 30 ms, TR = 2000 ms, flip angle (FA) = 90°, number of slices = 33, slice thickness = 4 mm, matrix size = 64 × 64, and field of view (FOV) = 220 × 220 mm.

### 2.5 Image preprocessing

Preprocessing of the resting‐state fMRI data was conducted using Data Processing Assistant for Resting‐State fMRI (DPABI 3.0),^20^ which is based on Statistical Parametric Mapping (SPM12). The first 10 images were discarded to ensure the inclusion of steady‐state data only. The remaining images underwent correction for slice timing and head motion, and head motion parameters were recorded after alignment correction. Participants with maximum displacement >2 mm in any plane, >2° of angular motion, or >0.2 mm in mean framewise displacement (FD) were excluded from further analysis.^21^ Subsequently, the motion‐corrected images were spatially normalized to a standard Montreal Neurological Institute (MNI) echo planar imaging (EPI) template, resliced to a voxel size of 3 × 3 × 3 mm^3^, and underwent linear detrending. To reduce the impact of low‐frequency drifts and high‐frequency noise, a bandpass filter (0.01 Hz < *f* < 0.1 Hz) was applied. Nuisance signals, including the signals of white matter and cerebrospinal fluid, were removed from the data, and the Friston‐24 parameters of head motion (6 head motion parameters, 6 head motion parameters at one time point before, and the 12 corresponding squared items) were regressed out from each time series.

### 2.6 Definition of region of interest

In accordance with prior research on habenular FC,^11^, ^22^ we selected two spherical seed regions of interest (ROIs) with a radius of 3 mm centered on two MNI coordinates: the left and right habenula [MNI (*x*, *y*, *z*): left = −2.8, −24.2, 2.3; right = 4.8, −24.1, 2.2].

### 2.7 Analyses of static and dynamic FC of the habenula

We performed a seed‐based whole‐brain approach to examine static functional connectivity (sFC) of the bilateral habenula using DPABI. The seed point reference time series of each habenular region was calculated by extracting and averaging the time series over all voxels within itself. Individual sFC maps of the bilateral habenula were generated by calculating Pearson's correlation coefficients between the mean time series of the ROIs and the time series of each voxel in the whole brain. Then, we transformed the correlation maps to *z* value maps using Fisher's *r*‐to‐*z* transformation. Finally, spatial smoothing with a Gaussian kernel of 6 mm full width at half maximum (FWHM) was applied for sFC calculation to decrease noise.

To assess dynamic functional connectivity (dFC), a sliding‐window approach was employed using the Temporal Dynamic Analysis (TDA) toolkits included in the DPABI software. A Hamming window was applied to the whole‐brain BOLD signals, and a sliding window size of 50 TRs and a window step of 1 TR were chosen for evaluating the whole‐brain dFC variability. The minimum window length should be no less than 1/*f* min (1/0.01 s = 100 s) according to previous studies^23^, ^24^; *f* min was defined as the minimum frequency of the time series. Additionally, other window lengths (30 TRs and 70 TRs) and shifting steps (1 TR) were tested to further examine their possible effects on the dFC results (shown in the Figures S1–S6). For each sliding window, correlation maps were computed by correlating the truncated time series of the habenula seeds with all other voxels, resulting in 181 sliding window correlation maps for each participant. These maps were then converted to *z* value maps using Fisher's *r‐*to*‐z* transformation to improve the normality of the correlation distribution. Then, the dFC maps were computed by calculating the standard deviation of 181 sliding‐window *z* value maps, and *Z* standardization was applied to the dFC maps. Finally, all the dFC maps were smoothed using a 6 mm FWHM Gaussian kernel.

### 2.8 Statistical analyses

Demographic and clinical data were analyzed using SPSS 25.0 (SPSS). Two‐sample *t*‐tests were used to compare the demographic information and neuropsychological scores between the LLD and HC groups, while a chi‐squared test was employed to compare the sex ratios. All tests were two‐tailed, and the significance level was set at a *p* value less than 0.05. Mediation analyses were conducted to explore the relationship between group differences between LLD patients and HCs (independent variable) and various cognitive scores (dependent variable), with the depressive scores (HAMD total scores and 5‐factor scores, GDS score) as mediators and age, sex and years of education as covariates. The mediation model was constructed using PROCESS 3.4.1 in SPSS, with a 95% confidence interval for the output and 5000 bootstrap samples. The one‐sample *t*‐test was performed to detect within‐group FC variability in each ROI in patients with LLD and HCs. To further examine the difference in FC patterns between LLD patients and HCs, a two‐sample *t*‐test was performed on the *z* value of each voxel within the union mask of the one‐sample *t*‐test results of the two groups. Age, sex, years of education and FD were included as nuisance covariates in the comparisons. Cluster‐level correction for multiple comparisons was conducted based on Gaussian random field (GRF) theory (voxel‐level *p* value <0.005, cluster‐level *p* value <0.05).

Partial correlation analyses were conducted to explore the relationship between neuropsychological scores and values of FC for each significant region while controlling for age, sex, FD, and years of education. False discovery rate (FDR) correction was used to correct the correlation analyses. Additionally, mediation analyses were conducted to explore the relationships between depressive scores (independent variable) and cognitive scores (dependent variable), with the FC values as mediators and age, sex, FD, and years of education as covariates. The mediation model was computed using PROCESS 3.4.1 in SPSS, with a 95% confidence interval for the output and 5000 bootstrap samples.

## 3 RESULTS

### 3.1 Demographic, clinical, and neuropsychological information

The demographic, clinical, and neuropsychological information of the LLD group and the HC group is listed in Table 1. No significant difference was found in age or sex ratios between the LLD group and HC group (*p* > 0.05), but the LLD group had fewer years of education and higher depressive scores (HAMD and GDS scores) than the HC group (*p* < 0.05). Regarding cognitive scores, significant differences were found in all assessments between the LLD group and the HC group (*p* < 0.05).

**TABLE 1:** Demographic and clinical data of participants.

Parameter	LLD (*n* = 127)	HCs (*n* = 75)	*χ* ^2^/*t*	*p* Value (two‐tailed)
Sex (Male/Female)	26/101	24/51	3.364	0.067^a^
Age (year)	67.65 ± 7.00	66.83 ± 5.86	0.860	0.391^b^
Education (year)	8.70 ± 4.04	10.92 ± 2.39	−4.33	<0.001^b^
Depression				
HAMD‐17	8.88 ± 7.17	1.47 ± 1.87	11.04	<0.001^b^
Psychomotor retardation (14 points)	2.37 ± 2.32	0.12 ± 0.40	10.49	<0.001^b^
Cognitive impairment (12 points)	0.96 ± 1.50	0.05 ± 0.23	6.67	<0.001^b^
Anxiety/somatization (18 points)	3.22 ± 3.13	0.43 ± 0.98	9.33	<0.001^b^
Sleep disturbance (6 points)	2.14 ± 1.93	0.85 ± 1.24	5.78	<0.001^b^
Weight (2 points)	0.20 ± 0.54	0.01 ± 0.12	3.85	<0.001^b^
GDS	5.20 ± 3.83	1.36 ± 1.42	9.36	<0.001^b^
Cognitive functions				
Global cognitive				
MMSE	23.20 ± 4.95	27.28 ± 1.42	−8.70	<0.001^b^
Memory				
AVLT‐Immediate recall	16.16 ± 5.97	20.83 ± 4.49	−6.19	<0.001^b^
AVLT‐Short‐term delayed recall	5.13 ± 2.78	7.57 ± 1.69	−7.61	<0.001^b^
AVLT‐Long‐term delayed recall	4.23 ± 3.20	7.03 ± 2.04	−7.44	<0.001^b^
AVLT‐Recognition	4.11 ± 3.20	6.98 ± 1.92	−7.22	<0.001^b^
WMT	3.70 ± 2.53	7.24 ± 2.78	−8.32	<0.001^b^
Information processing speed				
SDMT (s)	26.23 ± 11.10	37.20 ± 8.92	−7.12	<0.001^b^
Stroop A (s)	35.27 ± 13.45	28.62 ± 7.39	4.09	<0.001^b^
TMT A	64.80 ± 26.82	44.0 ± 12.51	5.88	<0.001^b^
Executive function				
TMT B	85.89 ± 35.91	57.65 ± 17.30	5.93	<0.001^b^
Stroop C (s)	96.71 ± 36.15	77.66 ± 21.99	4.17	<0.001^b^
Language				
BNT	19.95 ± 3.51	23.30 ± 2.26	−7.49	<0.001^b^
VFT	8.76 ± 4.39	12.05 ± 3.82	−5.52	<0.001^b^
Visuospatial skill				
ROCF	23.42 ± 6.87	28.54 ± 3.01	−6.64	<0.001^b^

Abbreviations: AVLT, Auditory Verbal Learning Test; BNT, Boston Naming Test; GDS, Geriatric Depression Scale; HAMD‐17, 17‐item Hamilton Depression Rating Scale; HCs, healthy controls; LLD, late‐life depression; MMSE: Mini‐mental State Examination; ROCF: Rey‐Osterrieth Complex Figure; SDMT, Symbol Digit Modalities Test; Stroop, Stroop Color and Word Test; TMT, Trail‐Making Test; VFT, Verbal Fluency Test; WMT, Working Memory Test.

^a^ *χ* ^2^ test.

^b^ Two‐sample *t*‐test.

### 3.2 Mediating effect of depressive symptoms on cognitive impairment

**FIGURE 1:**
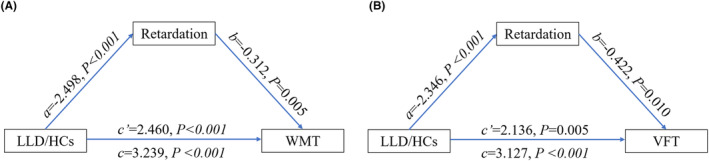
Associations between group difference and cognitive impairment were mediated by the depressive symptom. (A) The psychomotor retardation mediated the difference in WMT scores of LLD/HCs groups; (B) the psychomotor retardation mediated the difference in VFT scores of LLD/HCs groups. *a*, The effect of the independent variable on the mediating variable. *b*, the effect of the mediating variable on the dependent variable. *c*, the total effect of the independent variable on the dependent variable. *c*′, the direct effect of the independent variable on the dependent variable. HCs, healthy controls; LLD, late‐life depression; VFT, Verbal Fluency Test; WMT, Working Memory Test.

According to the mediation analyses, the HAMD psychomotor retardation score partially mediated the group difference (LLD/HC) in WMT and VFT (Figure 1). No other mediating effect of HAMD scores was observed on the group difference (LLD/HC) in cognitive scores (*p* > 0.05).

### 3.3 Comparison of sFC and dFC with the habenula as the seed

**FIGURE 2:**
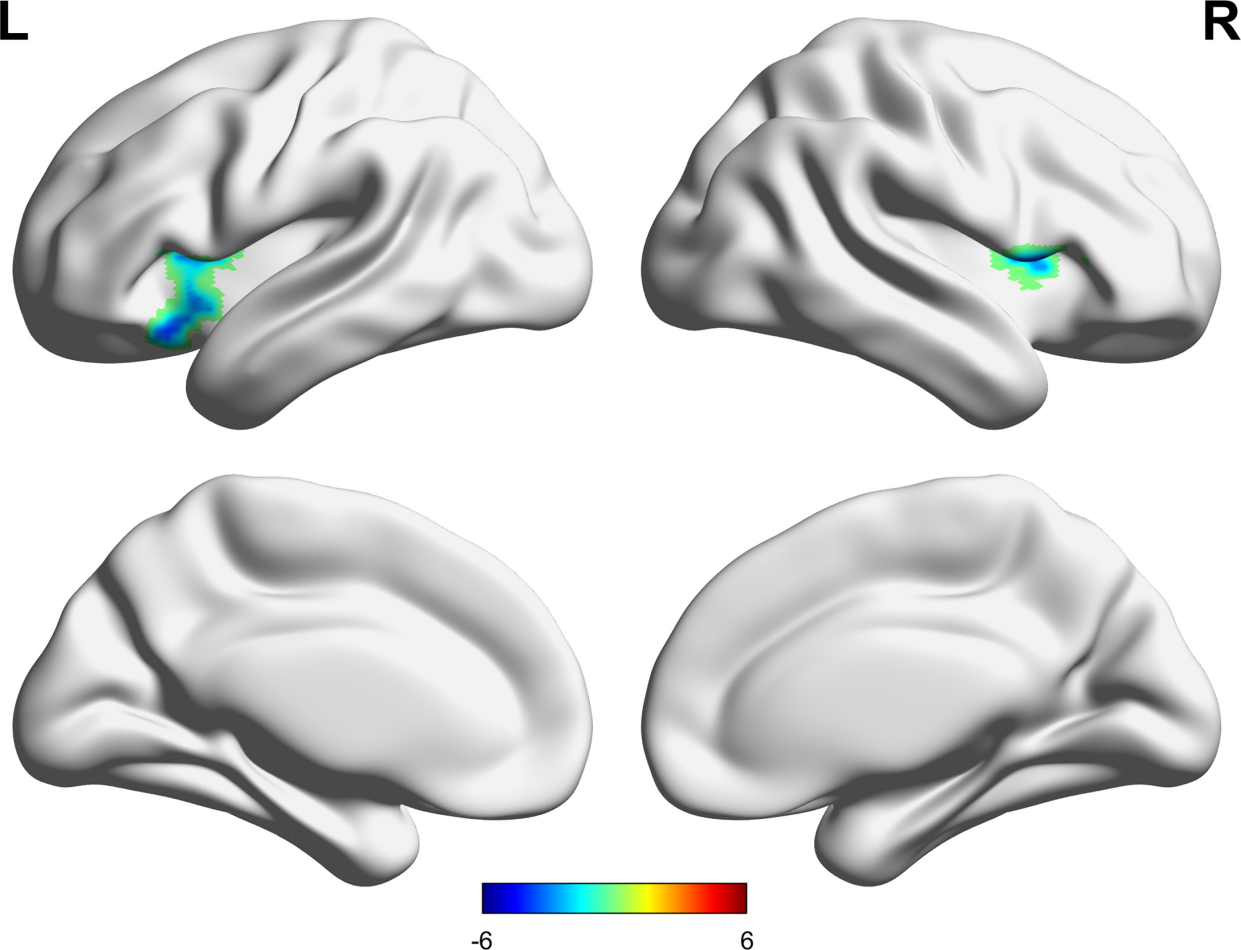
Comparison of the sFC of right habenula seed between the LLD group and the HC group (voxel *p* < 0.005, cluster *p* < 0.05, GRF corrected). The color bar indicates the *t* values from two‐sample *t*‐test analysis. GRF, Gaussian random field; HCs, healthy control; L (R), left (right) hemisphere; LLD, late‐life depression; sFC, static functional connectivity.

Compared with the HC group, the LLD group exhibited lower sFC between the right habenula and bilateral inferior frontal gyrus (IFG) (Table 2, Figure 2). When the left habenula was set as the seed, no significant difference in sFC was found between the LLD and HC groups.

**TABLE 2:** The areas of significantly different sFC between the LLD patients and the HCs (voxel *p* < 0.005, cluster *p* < 0.05, GRF corrected).

Seeds	Regions	Brodmann area	Peak MNI			Peak *t* value	Cluster size
			*X*	*Y*	*Z*		
Right habenula	Left IFG, orbital part	47	−33	21	−15	−4.02	130
	Right IFG, opercular part	48	42	12	6	−5.48	102

Abbreviations: GRF, Gaussian random field; HCs, healthy controls; IFG, inferior frontal gyrus; LLD, late‐life depression; MNI, Montreal Neurological Institute Coordinates; sFC, static functional connectivity.

There was no significant difference in the dFC of the left and right habenula between the LLD and HC groups when various window lengths were applied (50 TRs, 30 TRs and 70 TRs) (uncorrected results are shown in the Figures S1–S6).

### 3.4 Correlation analyses

**FIGURE 3:**
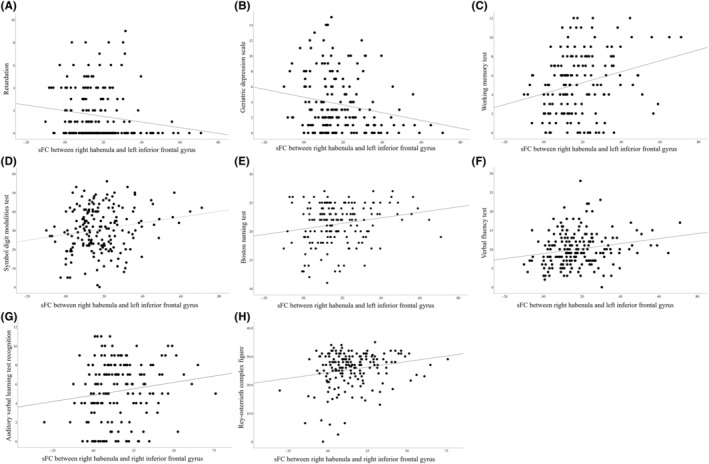
The correlation between abnormal sFC values and neuropsychological scores. The sFC between right habenula and left IFG was negatively associated with scores of psychomotor retardations (*r* = −0.146, *p =* 0.043, corrected *p =* 0.043) (A), GDS (*r* = −0.191, *p =* 0.013, corrected *p =* 0.026) (B), and was positively associated with scores of WMT (*r* = 0.238, *p =* 0.002, corrected *p =* 0.017) (C), SDMT (*r* = 0.199, *p =* 0.007, corrected *p =* 0.049) (D), BNT (*r* = 0.156, *p =* 0.047, corrected *p =* 0.047) (E) and VFT (*r* = 0.222, *p =* 0.002, corrected *p =* 0.017) (F).The sFC between right habenula and right IFG was positively associated with scores of AVLT recognition (*r* = 0.165, *p =* 0.036, corrected *p =* 0.036) (G) and ROCF (*r* = 0.200, *p =* 0.010, corrected *p =* 0.049) (H). IFG, inferior frontal gyrus; sFC, static functional connectivity.

The sFC between the right habenula and left IFG was negatively associated with HAMD psychomotor retardation scores (*r* = −0.146, *p =* 0.043, corrected *p =* 0.043) (Figure 3A) and GDS scores (*r* = −0.191, *p =* 0.013, corrected *p =* 0.026) (Figure 3B) but was positively associated with WMT scores (*r* = 0.238, *p =* 0.002, corrected *p =* 0.017) (Figure 3C), SDMT scores (*r* = 0.199, *p =* 0.007, corrected *p =* 0.049) (Figure 3D), BNT scores (*r* = 0.156, *p =* 0.047, corrected *p =* 0.047) (Figure 3E), and VFT scores (*r* = 0.222, *p =* 0.002, corrected *p =* 0.017) (Figure 3F).

The sFC between the right habenula and right IFG was positively associated with AVLT recognition scores (*r* = 0.165, *p =* 0.036, corrected *p =* 0.036) (Figure 3G) and ROCF scores (*r* = 0.200, *p =* 0.010, corrected *p =* 0.049) (Figure 3H).

### 3.5 Mediating effect of habenular sFC on the relationship between depressive symptoms and cognitive impairment

**FIGURE 4:**
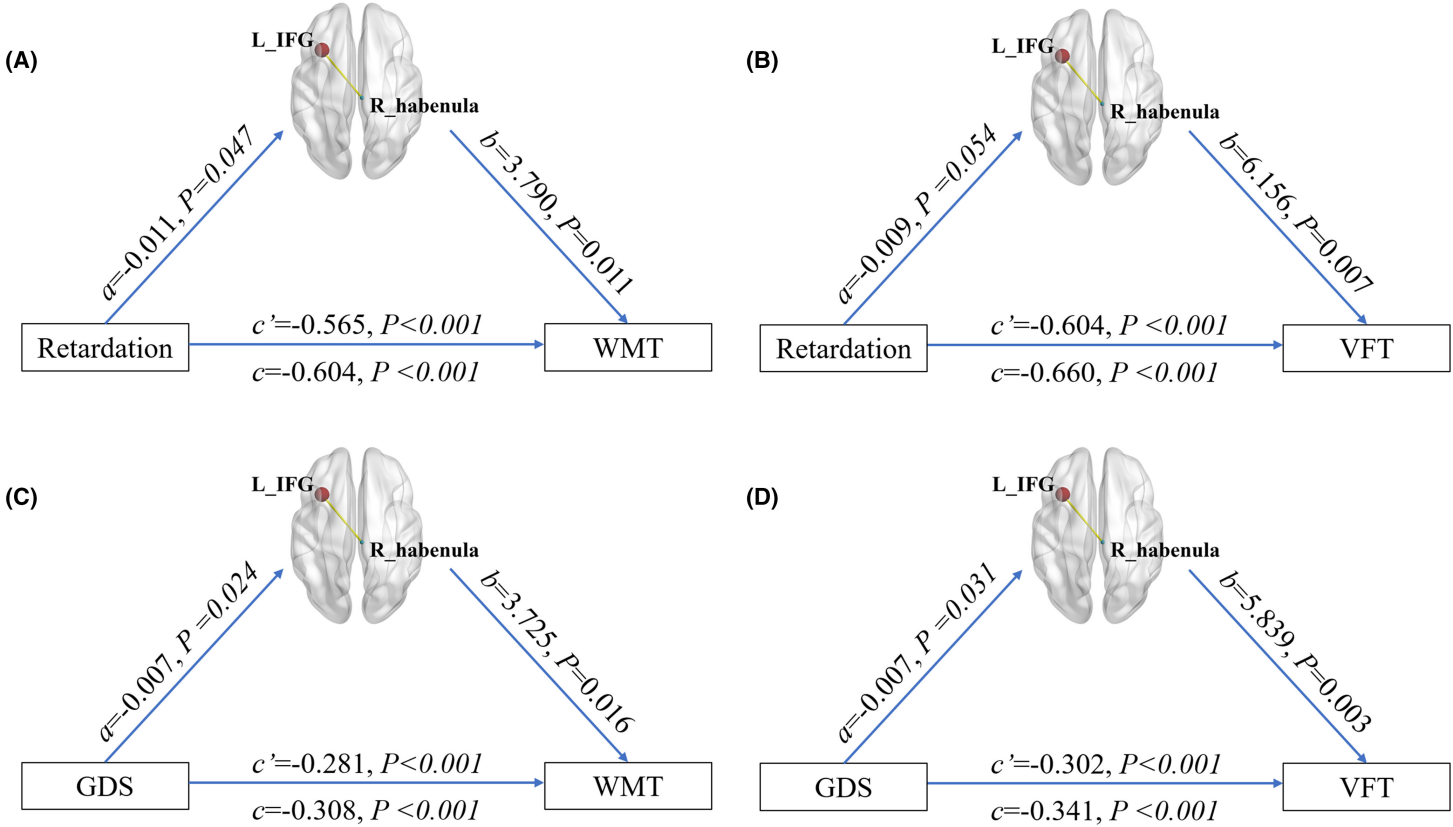
Associations between depressive symptom and cognitive impairment were mediated by the sFC between right habenula and left IFG. The sFC between right habenula and left IFG exhibited partially mediated effect on ①the relationship between psychomotor retardation scores and WMT score (A); the relationship between psychomotor retardation scores and VFT score (B); the relationship between GDS scores and WMT score (C); the relationship between GDS scores and VFT score (D). *a*, The effect of the independent variable on the mediating variable. *b*, The effect of the mediating variable on the dependent variable. *c*, The total effect of the independent variable on the dependent variable. *c*′, The direct effect of the independent variable on the dependent variable. GDS, Geriatric Depression Scale; IFG, inferior frontal gyrus; L (R), left (right) hemisphere; sFC, static functional connectivity; VFT, Verbal Fluency Test; WMT, Working Memory Test.

There were four significant models in the mediation analyses as follows: the sFC between the right habenula and left IFG exhibited a partial mediating effect on ① the relationship between psychomotor retardation scores and WMT scores (Figure 4A), ② the relationship between psychomotor retardation scores and VFT scores (Figure 4B), ③ the relationship between GDS scores and WMT scores (Figure 4C), and ④ the relationship between GDS scores and VFT scores (Figure 4D).

No significant mediating effect of the sFC between the right habenula and right IFG on the association between the depressive scores and cognitive variables was observed, and no significant mediating effect of the other dFC of the habenula was observed (*p* > 0.05).

## 4 DISCUSSION

To the best of our knowledge, this is the first study to report disrupted FC of the habenula in LLD, which highlights potential similarities in neural mechanisms across age groups and enhances understanding of neural connectivity in MDD and LLD patients. The main results were as follows. First, more severe depressive symptoms (higher GDS scores and HAMD psychomotor retardation scores) led to greater cognitive impairment (language and working memory) in patients with LLD. Second, compared with the controls, patients with LLD exhibited decreased sFC between the right habenula and bilateral IFG, but there was no significant difference in dFC between the two groups. Third, the decreased sFC between the right habenula and IFG was associated with more severe depressive symptoms and cognitive impairment (language, memory, and visuospatial skills). Fourth, the sFC between the right habenula and left IFG mediated the relationship between depressive symptoms (higher GDS score and HAMD psychomotor retardation score) and cognitive impairment (language and working memory).

The present study showed that patients with LLD exhibited decreased sFC between the habenula and IFG, which is consistent with the disrupted FC between the habenula and the frontal cortex (middle frontal gyrus and frontal pole) in patients with MDD reported in previous studies.^9^, ^12^ The IFG is involved in the perception and interpretation of emotional stimuli, emotion regulation, empathy and emotional perspective‐taking,^25^ and dysfunction of the IFG in patients with depression has been reported in previous studies.^26^ Therefore, the decreased sFC between the habenula and IFG indicates a functional disconnection of these two important hubs of emotion regulation in patients with LLD. Additionally, the decreased sFC between the habenula and IFG was negatively associated with the severity of depressive symptoms (especially psychomotor retardation) but not cognitive bias, anxiety/somatization, sleep disturbance or weight, which provides more detailed information about the aspect of depressive symptoms affected by habenular FC. It has been shown that stimulating the habenula may alleviate depressive symptoms,^6^ but the deep location of the habenula makes such interventions difficult. Considering that the effect of neuromodulation targeting the FC between the frontal cortex and deep regions on depression has been reported by an increasing number of studies^27^ and that the IFG is a superficial and accessible target,^28^ our results have important implications as they indicate potential targets for alleviating depressive symptoms in LLD patients; however, further clinical trials are needed.

Interestingly, there was no significant difference in habenular dFC between LLD patients and controls, even when different sliding‐window lengths were applied (30, 50, and 70 s). sFC and dFC are different approaches used to investigate the relationships and interactions of brain regions: sFC is based on the premise that the connections between brain regions remain consistent over time and reflect their average connectivity throughout the entire fMRI session, and dFC is based on the assumption that connections between regions change over time and exhibit temporal variability in the spontaneous fluctuations of connectivity.^29^ Therefore, the current results suggest that the abnormal connectivity between the habenula and IFG involves reductions in connectivity strength but not in stability. However, in addition to the sliding‐window method, there are other methods used to analyze dFC (such as hidden Markov models)^30^ that may generate different results; thus, the present findings need to be interpreted with caution.

In addition to its association with depressive symptoms, the decreased sFC between the habenula and IFG exhibited a small but significant association with cognitive impairment in patients with LLD, including language, memory, and visuospatial skills. The habenula has been shown to be involved in working memory^29^ and spatial memory processes,^31^ and a disrupted connection between the habenula and frontal cortex causes working memory deficits,^29^ which may result from the connectivity of the habenula with brain areas implicated in cognitive processing and regulation of the neurotransmitter system, which is critical for cognitive processing. Similar to the habenula, the IFG is involved in various cognitive domains, such as language processing, working memory, cognitive control, and response inhibition.^32^ Therefore, the present results indicate that the disconnection of the habenula and IFG may be involved in the pathological mechanism underlying the overall cognitive impairment in patients with LLD. Considering that LLD is a risk factor for dementia,^3^ the decreased sFC between the habenula and IFG may also represent a potential biomarker for LLD that may facilitate early identification of high‐risk individuals, but this needs to be verified by longitudinal studies.

The present study revealed that psychomotor retardation was partially associated with deficits in working memory and language in patients with LLD, which is consistent with previous evidence that more severe depressive symptoms resulted in worse cognitive performance. Interestingly, the present study also revealed that the sFC between the right habenula and left IFG partially mediated the relationship between psychomotor retardation and cognitive impairment, specifically in the working memory and language domains. It remains unclear how this mediating effect occurs, and we hypothesized that more severe depressive symptoms may represent more stress‐related pathological processes, which leads to hyperactivity of the habenula and its disconnection from the IFG, causing cognitive impairment. Regarding working memory, the current result is consistent with previous evidence that both the habenula and IFG are associated with working memory,^29^, ^33^ and it is reasonable that depressive symptoms may disrupt the connection between these two regions and lead to working memory impairment. Regarding the language domain, the IFG is crucial for language production and comprehension,^34^ but there is little evidence that the habenula plays a role in language processing. Therefore, the role of the habenula in language processing is likely to be indirect, and further research is needed to elucidate the exact mechanisms and pathways through which the habenula interacts with the IFG or other brain regions to influence language function. Overall, these results indicate that more severe depressive symptoms may impair cognitive function by disrupting the sFC between the right habenula and left IFG in elderly people, which complements literature about the potential mechanisms linking depression and cognitive impairment, provides a deeper understanding of their overlapping brain network, and contributes to the development of more targeted and effective interventions in patients with LLD.

## 5 LIMITATIONS

The present findings should be interpreted in light of several limitations. First, the present study had a cross‐sectional design, and it remains unclear how the decreased sFC between the habenula and IFG mediated the relationship between cognitive impairment and depressive symptoms. The relationships among cognition, depression and habenular FC need to be explored in future studies. Second, sFC reflects the correlation between the activity of two regions (the habenula and the frontal cortex), and further analyses involving Granger causality or dynamic causal modeling could clarify how these regions interact with each other. Third, the present study did not include biomarkers related to neuroplasticity, inflammation or cortisol, and the mechanism by which habenular sFC mediates the relationship between depressive symptoms and cognitive impairment in LLD remains unclear. Fourth, previous studies suggested that the lateral and medial parts of the habenula are different, and future studies applying MRI with higher resolution could provide a deeper understanding of their potentially different roles in LLD. Fifth, the presence of variation in antidepressant types and doses could potentially introduce a confounding element into the intricate interplay among FC, cognitive function, and depressive symptoms in patients with LLD. Sixth, years of education was included as a covariate in the statistical analyses. The lower education level of patients with LLD may have exerted a confounding effect, and the present results should be interpreted with caution.

## 6 CONCLUSION

Patients with LLD exhibited decreased sFC between the right habenula and bilateral IFG, and the decreased sFC between the right habenula and left IFG mediated the relationship between depressive symptoms (especially psychomotor retardation) and cognitive impairment (verbal fluency and working memory). The disconnection of the habenula and IFG altered the underlying pathway connecting depressive symptoms and cognitive impairment. Mapping the abnormal patterns of habenular FC in patients with LLD provided a more in‐depth understanding of the pathological mechanism and could inform the development of more targeted and effective therapeutic interventions, improving the management and treatment outcomes of LLD.

## AUTHOR CONTRIBUTIONS

TS and BC acquired the data, analyzed and interpreted the data, and drafted the manuscript. MY and XZ designed and conceptualized the study, analyzed and interpreted the data, and critically revised the manuscript. QW, MZ, HZ, ZW, and GL acquired the data and critically revised the manuscript. XZ and YN critically revised the manuscript. All authors read and approved the final manuscript.

## FUNDING INFORMATION

This study was supported by a grant from the National Natural Science Foundation of China (No. 82101508), the Guangzhou Municipal Psychiatric Diseases Clinical Transformation Laboratory (No: 201805010009), the Key Laboratory for Innovation Platform Plan, the Science and Technology Program of Guangzhou, China, the Science and Technology Plan Project of Guangdong Province (No. 2019B030316001). Guangzhou Municipal Key Discipline in Medicine (2021–2023); Guangzhou High‐level Clinical Key Specialty; Guangzhou Research‐oriented Hospital. 2022 Student Innovation Ability Enhancement Project of Guangzhou Medical University (0‐2‐40‐8‐230‐4‐19083XM). The funders had no role in the study design, data collection and analysis, decision to publish or preparation of the manuscript.

## CONFLICT OF INTEREST STATEMENT

The authors have no actual or potential conflicts of interest to declare.

## Supporting information


Figure S1

## Data Availability

The datasets used or analyzed during the current study are available from the corresponding author on reasonable request.
