# Supplementary material for: Disrupted functional connectivity of the habenula links psychomotor retardation and deficit of verbal fluency and working memory in late‐life depression
Source: CNS Neurosci Ther. 2023 Oct 7;30(4):e14490. doi: 10.1111/cns.14490 (PMC11017447; doi:10.1111/cns.14490)
Supplement: Supplementary file 1 — Figure S1 [file CNS-30-e14490-s001.docx]

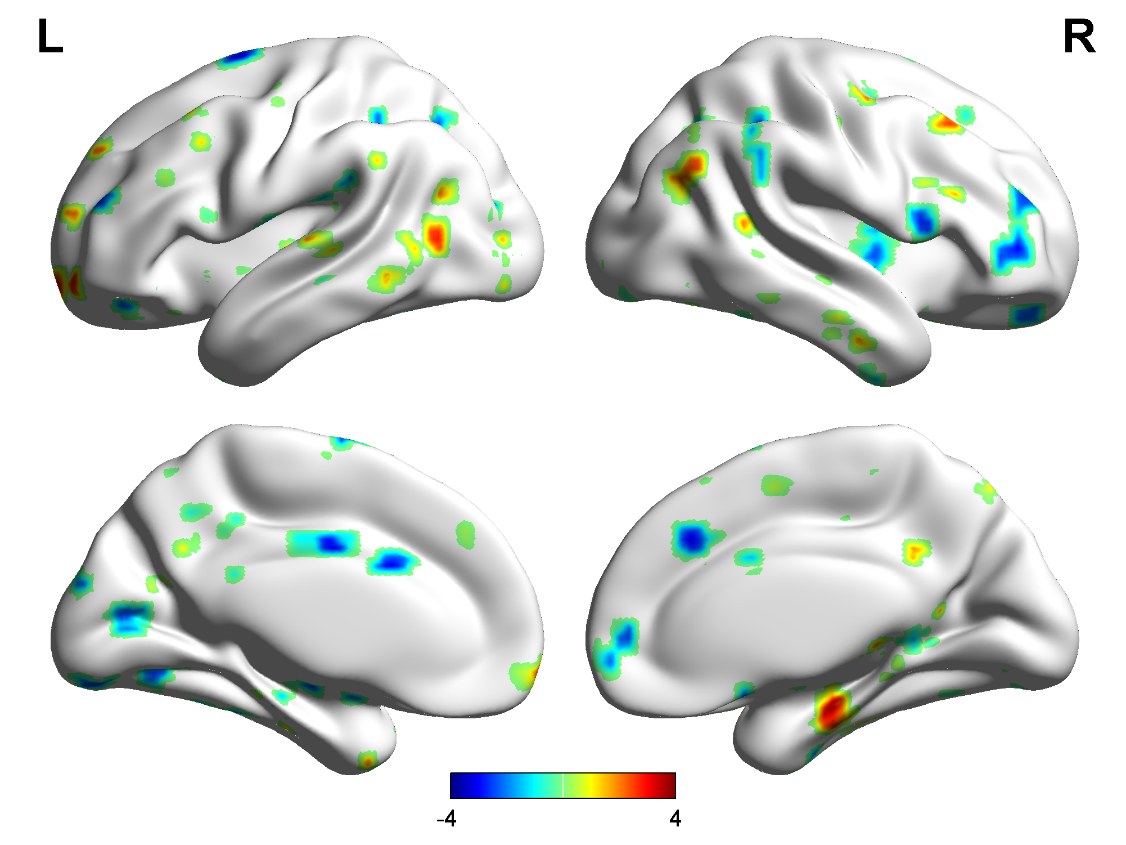


**Figure S1. Comparison of the dFC of left habenula between the LLD group and the HC group (windows lengths 30 TRs, uncorrected *p* < 0.05).** The color bar indicates the *t* values from two-sample *t* test analysis. dFC, dynamic functional connectivity; L (R), left (right) hemisphere.


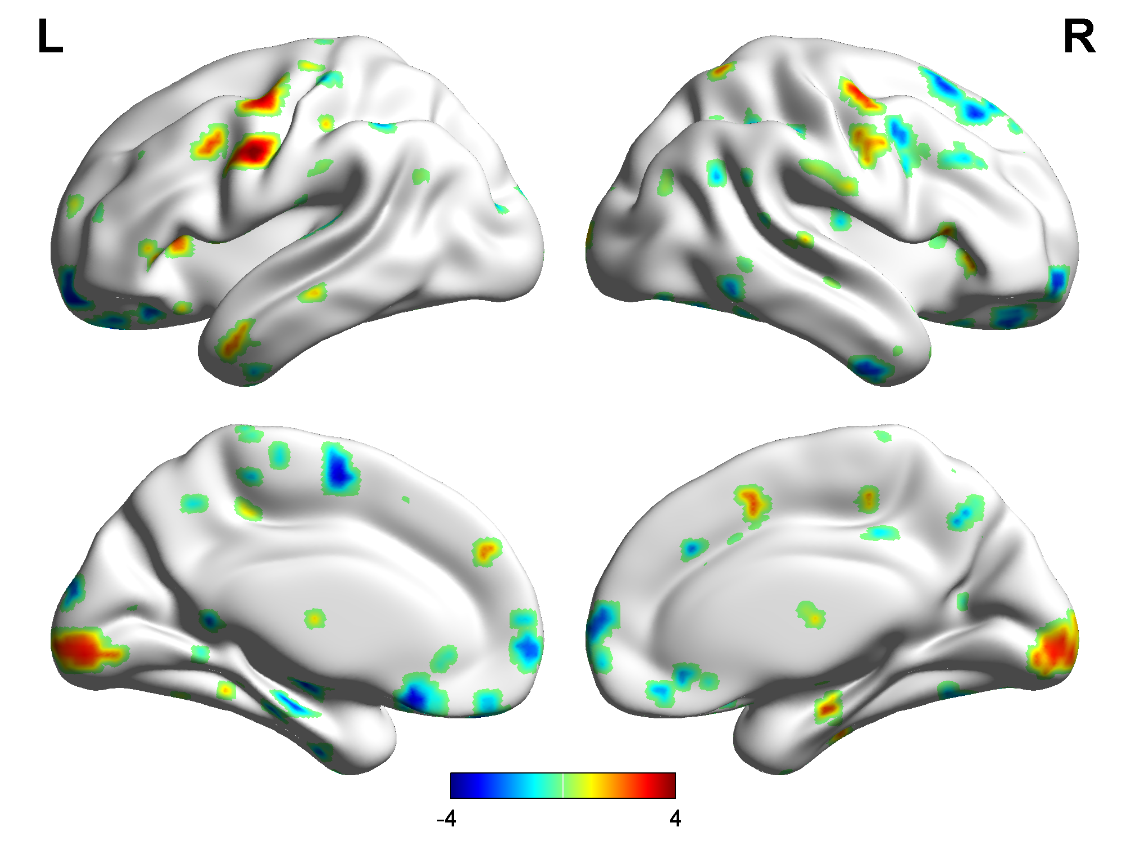


**Figure S2. Comparison of the dFC of right habenula between the LLD group and the HC group (windows lengths 30 TRs, uncorrected *p* < 0.05).** The color bar indicates the *t* values from two-sample *t* test analysis. dFC, dynamic functional connectivity; L (R), left (right) hemisphere.


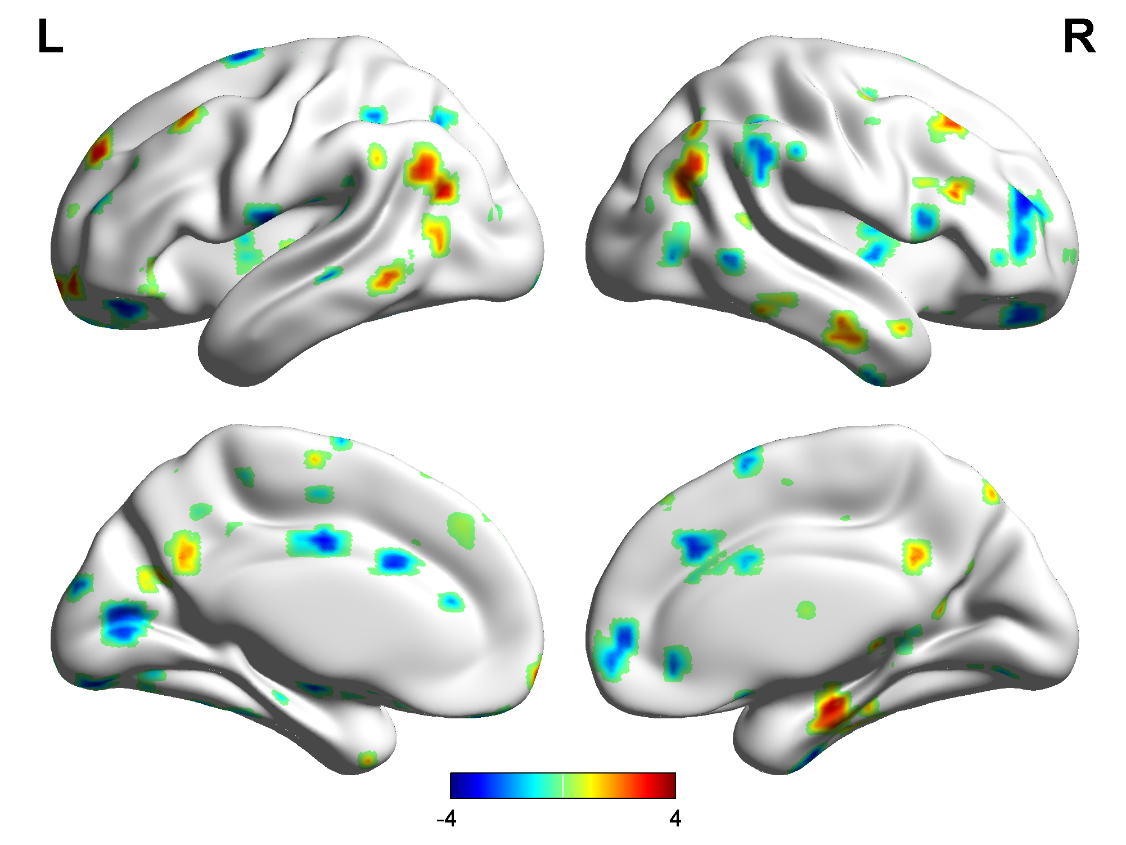


**Figure S3. Comparison of the dFC of left habenula between the LLD group and the HC group (windows lengths 50 TRs, uncorrected *p* < 0.05).** The color bar indicates the *t* values from two-sample *t* test analysis. dFC, dynamic functional connectivity; L (R), left (right) hemisphere.


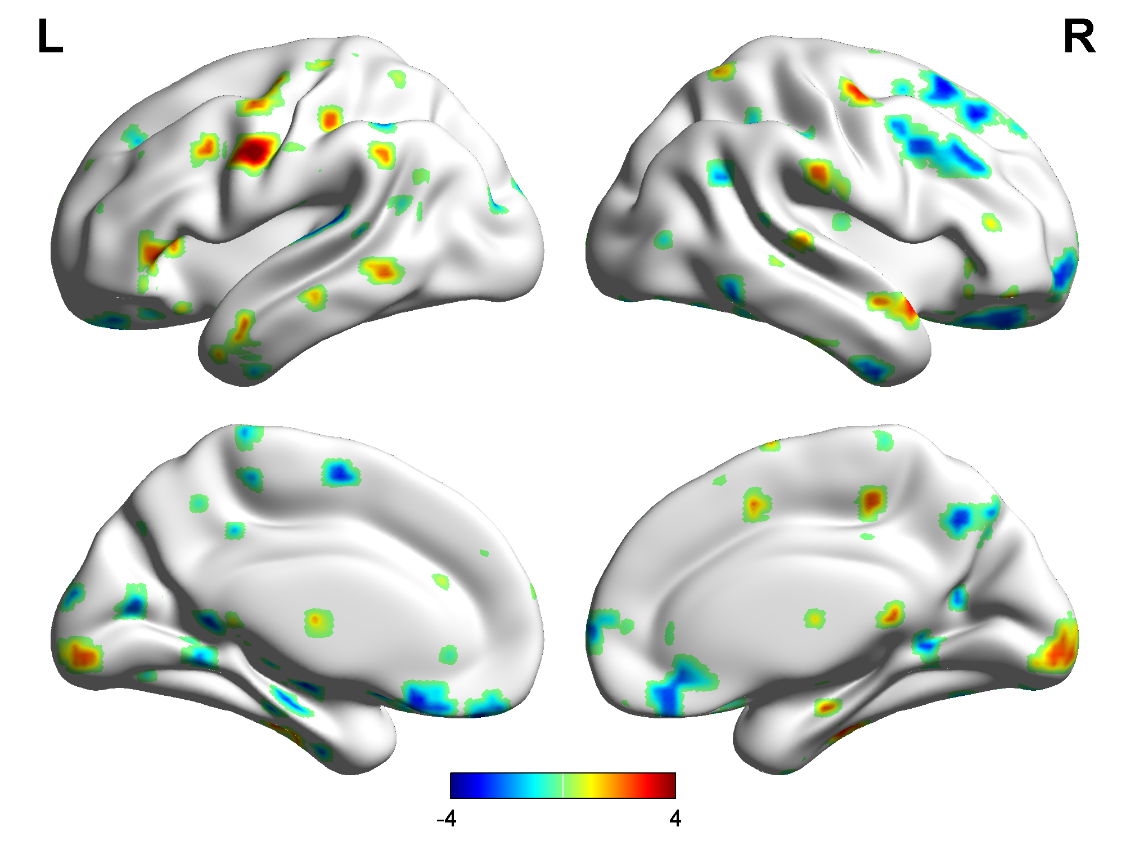


**Figure S4. Comparison of the dFC of right habenula between the LLD group and the HC group (windows lengths 50 TRs, uncorrected *p* < 0.05).** The color bar indicates the *t* values from two-sample *t* test analysis. dFC, dynamic functional connectivity; L (R), left (right) hemisphere.
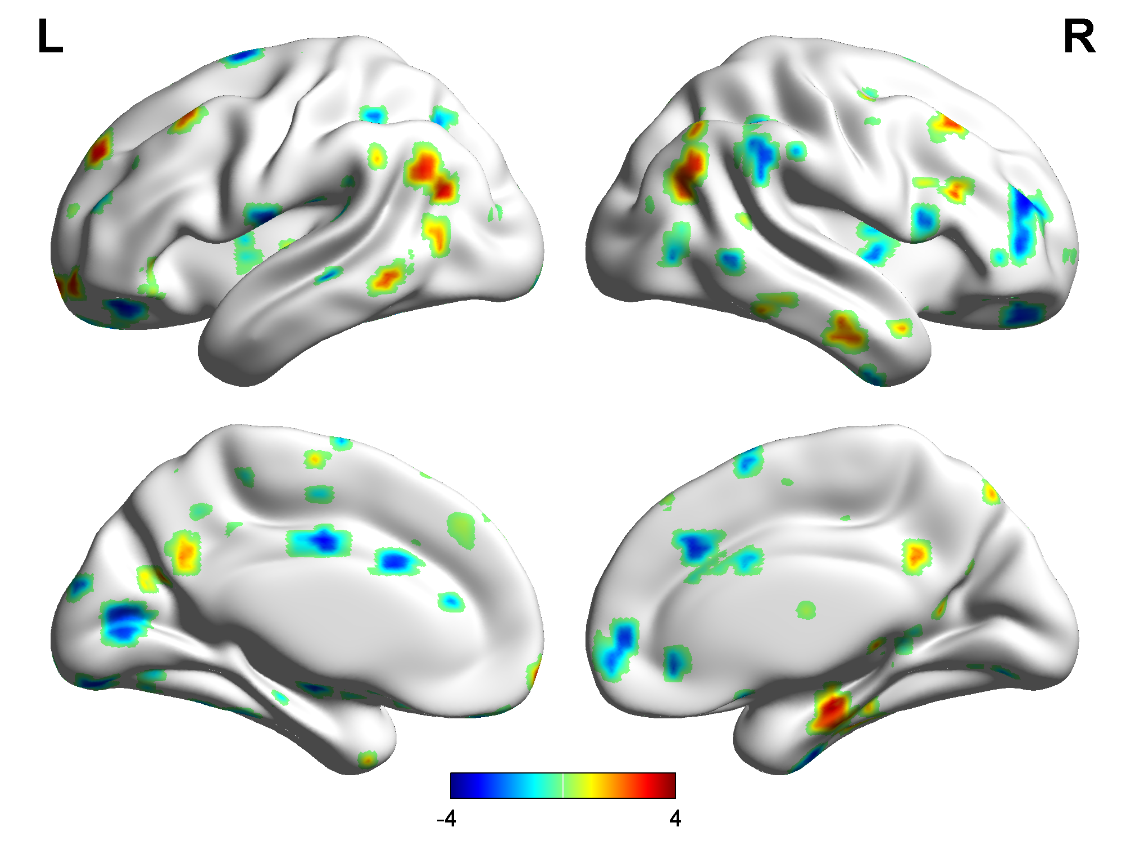


**Figure S5. Comparison of the dFC of left habenula between the LLD group and the HC group (windows lengths 70 TRs, uncorrected *p* < 0.05).** The color bar indicates the *t* values from two-sample *t* test analysis. dFC, dynamic functional connectivity; L (R), left (right) hemisphere.


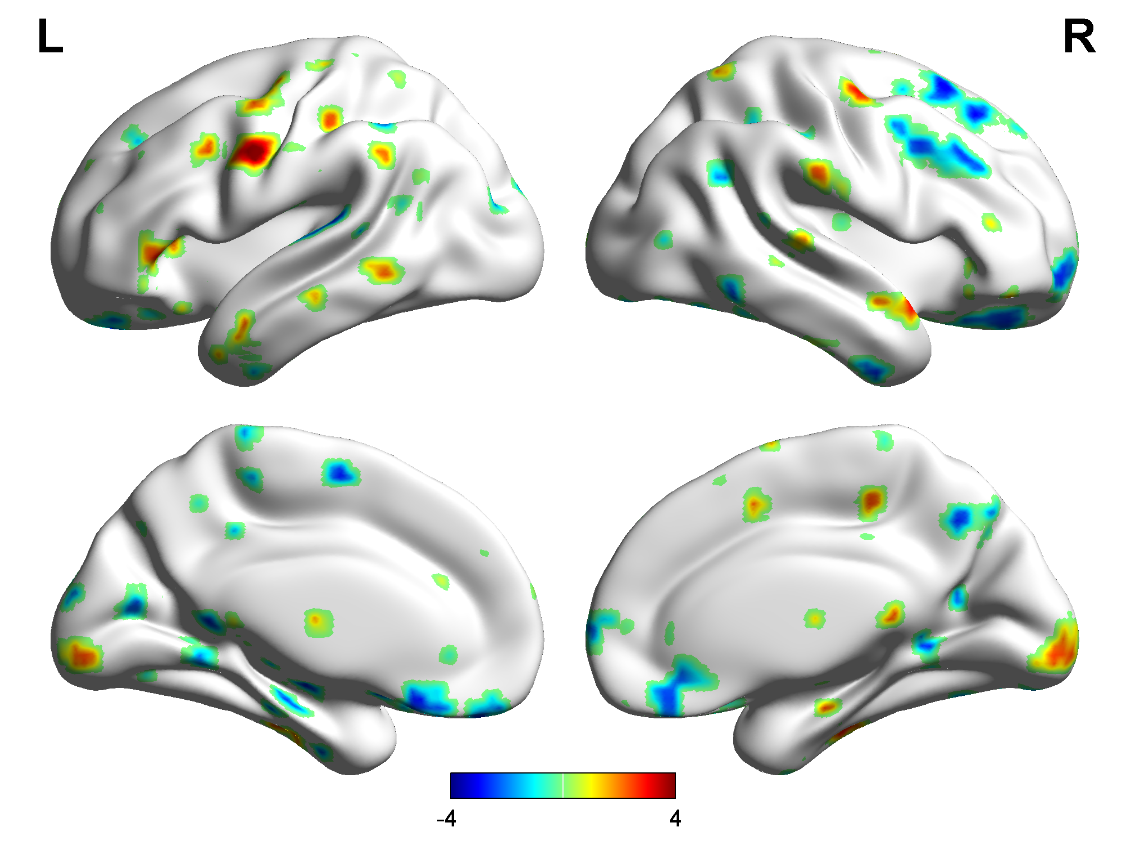


**Figure S6. Comparison of the dFC of right habenula between the LLD group and the HC group (windows lengths 70 TRs, uncorrected *p* < 0.05).** The color bar indicates the *t* values from two-sample *t* test analysis. dFC, dynamic functional connectivity; L (R), left (right) hemisphere.
